# Blood Pressure and Renal Responses to Orthostatic Stress Before and After Radiofrequency Renal Denervation in Patients with Resistant Hypertension

**DOI:** 10.3389/fcvm.2018.00042

**Published:** 2018-05-23

**Authors:** Yann Vuignier, Eric Grouzmann, Olivier Muller, Nima Vakilzadeh, Mohamed Faouzi, Marc P. Maillard, Salah D. Qanadli, Michel Burnier, Grégoire Wuerzner

**Affiliations:** ^1^Service of Nephrology and Hypertension, Department of Medicine, Lausanne University Hospital, Lausanne, Switzerland; ^2^Laboratoire des Catécholamines et Peptides, Service de Biomédecine, Lausanne University Hospital, Lausanne, Switzerland; ^3^Department of Cardiology, Lausanne University Hospital, Lausanne, Switzerland; ^4^Institute of Social and Preventive Medicine, Lausanne University Hospital, Lausanne, Switzerland; ^5^Cardio-Thoracic and Vascular Unit, Department of Radiology, Lausanne University Hospital, Lausanne, Switzerland

**Keywords:** renal denervation, lower body negative pressure, hypertension, sympathetic nervous system, glomerular filtration rate, renal plasma flow, sodium excretion

## Abstract

**Background/Aims:**

In patients with resistant hypertension, renal denervation (RDN) studies have mainly focused their outcomes on blood pressure (BP). The aim of this study was to evaluate the long-term effect of RDN on neurohormonal profiles, renal hemodynamics and sodium excretion in a resting state and during stress induced by lower body negative pressure (LBNP).

**Materials and methods:**

This was a single center prospective observational study. Norepinephrine, plasma renin activity (PRA), glomerular filtration rate (GFR), renal plasma flow (RPF) and sodium excretion were measured in unstimulated conditions (rest) and after one hour of LBNP at three different time points: before (M0), one (M1) and twelve months (M12) after RDN.

**Results:**

Thirteen patients with resistant hypertension were included. In the resting state, no differences were observed in norepinephrine, PRA, sodium excretion and mean BP levels after RDN. GFR (78 ± 32 ml/min at M0 vs 66 ± 26 ml/min at M12 (*p* = 0.012) and filtration fraction (22.6 ±5.4% at M0 vs 15.1 ±5.3% at M12 (*p* = 0.002)) both decreased after RDN. During LBNP, the magnitude of the mean BP increase was reduced from +6.8 ± 6.6 mm Hg at M0 to +2.3 ± 1.3 mm Hg at M12 (*p* = 0.005). The LBNP-induced increase in norepinephrine and decrease in GFR and sodium excretion observed before RDN were blunted after the procedure.

**Conclusion:**

A decrease in GFR and filtration fraction was observed one year after RDN. In addition, our results suggest that RDN blunts not only the norepinephrine but also the mean BP, the GFR and the sodium excretion responses to an orthostatic stress one year after the intervention.

**Registry number:**

NCT01734096

## Introduction

Catheter-based radiofrequency ablation of renal sympathetic nerves has been developed specifically for the treatment of resistant hypertension (1). However, its use remains controversial as the enthusiasm generated by the first publications reporting substantial reduction in office blood pressure (BP) was tempered by the heterogeneity of the BP response and by the results of the Symplicity HTN3 trial, which failed to demonstrate a significant difference in BP reduction between renal denervation (RDN) and a sham procedure (2, 3). Moderate but unpredictable reductions in office and ambulatory BP, however, have been demonstrated in subsequent controlled trials, which used either a strict standardized stepped-care antihypertensive treatment or an off medication design (4, 5).

The rationale for using the sympathetic nervous system (SNS) as a therapeutic target is the increased sympathetic tone observed in hypertension (6). This is particularly important at the level of the kidneys where the SNS contributes to the regulation of renin secretion, renal hemodynamic and sodium excretion (7). The objective of RDN is to destroy the sympathetic nerves fibers surrounding the renal arteries and therefore limit afferent and efferent signaling of the renal SNS. The procedure is non-selective as both afferent and efferent sympathetic nerves fibers can be affected. Today, the extent of renal denervation achieved cannot be assessed easily during or after the procedure in humans. The efficacy of the intervention has been judged almost exclusively on BP reduction, which is a rather indirect and imprecise way of assessing the effectiveness of the procedure. Yet office or out-of-office BP have been used as primary objective in most trials (1, 2, 4, 8). Independently of its effect on BP, RDN could affect renal plasma flow, renin release and sodium excretion, which are directly related to renal SNS activity (7). So far, few clinical studies have evaluated the impact of RDN on renal function other than estimated glomerular filtration rate (GFR).

In dogs, Di Bona et al have shown that progressive electric stimulation of renal sympathetic nerves causes an increase in plasma renin activity at low frequency, an increase in renal tubular sodium reabsorption at higher frequency and renal vasoconstriction at very high frequency (7). We have shown that in humans, kidneys react similarly to increasing levels of lower body negative pressure (LBNP), leading to similar stepwise increase in peripheral norepinephrine release, activity of the renin-angiotensin system, renal tubular reabsorption of sodium (9). During LBNP, venous blood is pooled in the lower extremities, thereby decreasing central venous pressure and unloading cardiopulmonary baroreceptors, which induces a reflex activation of the renin-angiotensin-aldosterone system (RAAS) and the SNS and depending on the magnitude of the negative pressure, affect cardiovascular and renal function (10, 11). In the present study, we hypothesized that if RDN was effective in disrupting the efferent renal sympathetic nervous pathway, changes in the plasma renin activity, renal tubular sodium excretion and renal plasma flow would be observed after RDN either in non-stimulated conditions (resting state) and particularly in response to an orthostatic stress induced by LBNP.

Therefore, the objectives of the study were to assess the long-term effect of RDN on the plasma norepinephrine and renin activity profile, renal hemodynamics and sodium excretion in standardized non-stimulated conditions and during an orthostatic stress induced by LBNP.

## Methods

The study was a single-center prospective observational study. The protocol was approved by the local Ethical Committee [Commission cantonale d'éthique de la recherche sur l'être humain (CER-VD)]. Written informed consent was obtained from all participants.

### Study Population

Patients were eligible for this study if they were referred for RDN and were older than 18 years. Patients were excluded from the study if they had orthostatic hypotension (positive Shellong’s test). The decision to perform RDN was taken by a multidisciplinary team including an interventional radiologist, a cardiologist and a hypertension specialist. RDN eligibility criteria were : (1) resistant hypertension defined as daytime ambulatory BP >135/85 mm Hg or nighttime BP >120/70 mm Hg despite the use of three antihypertensive drugs [one blocker of the renin-angiotensin system, one calcium channel blocker (CCB) or one beta blocker if CCB were not tolerated and one diuretic] at maximum tolerated doses (2) adequate adherence (>80%) to these 3 drugs assessed by the medication event monitoring system (MEMS®), (3) no secondary cause of hypertension (primary aldosteronism, hypothyroidism, Cushing syndrome, pheochromocytoma or renal artery stenosis), (4) estimated GFR > 30 ml/min/1.73 m^2^, (5) a suitable renal artery anatomy without extensive calcification (at least one renal artery on each side, renal artery main trunk's length >2 cm, renal artery diameter >4 mm). Radiofrequency renal denervation was performed under general anesthesia by one experienced interventional cardiologist and one experienced interventional radiologist using the Symplicity Flex catheter (Medtronic, MN, USA) or EnligHTN IV catheter (St Jude Medical, St Paul, MN). Both doctors were proctored before the initiation of the study. The target number of renal artery ablation points was five in each renal artery according to initial recommendations from manufacturers. The same antihypertensive treatment was continued after RDN. The antihypertensive treatment was reduced in case of hypotensive symptoms or if office systolic blood pressure was <120 mm Hg. Target office BP pressure was <140/90 mm Hg. Patients were seen in the outpatient hypertension clinic for screening and one week, three months, six months after RDN. Study days (renal clearance studies) were performed before RDN (M0), one month (M1) and 12 months (M12) after RDN. Recruitment started in 2012 and the last patient last study day was in May 2015.

### Data Collection

Patients included in the study were investigated on three separate standardized study days (M0, M1 and M12). On the day before the study days, a 24 h urine collection to estimate sodium intake and a 24 h ambulatory BP measurement (ABPM) with an adequately sized cuff (WatchBP03, Microlife AG, 9443 Widnau, Switzerland) were started. BP measurements during ABPM were taken every 20 min during the day and every 30 min at night. Daytime and nighttime period were defined according to patients' diary (bedtime and wakeup time) (12). Patients were studied with water and sodium intake ad libitum before the study day. Caffeine containing beverages and smoking was prohibited in the 24 h prior to the study day. In order to compare study days before and after RDN in standardized conditions, antihypertensive therapy was fixed to a combination of an angiotensin II receptor blocker and a calcium channel blocker in all patients in the morning of the study days. All other antihypertensive drugs were stopped 24 h before the study day.

**Figure 1:**
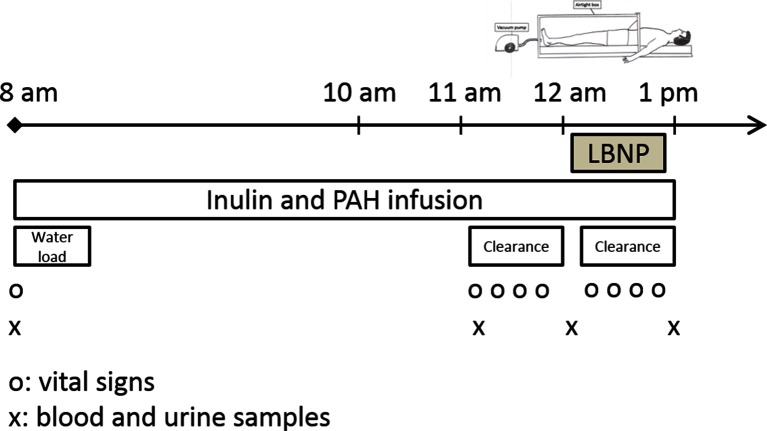
Study day schedule, which was repeated before renal denervation (M0), one month after renal denervation (M1) and 12 months after renal denervation (M12). LBNP, lower body negative pressure; PAH, para-amino hippurate. Infusion of inulin and PAH were used to measure glomerular filtration rate and renal plasma flow. LBNP was fixed at −30 mbar for one hour.

On study days (Figure 1), patients were asked to come to the clinic at 07.30 am. A venous catheter was inserted in each forearm: one for blood sampling and one for inulin (Inustest®, Fresenius Kabi Austria GmbH) and para-aminohippuric acid (PAH, Merck Sharp & Dhome Corp., USA) infusion. Inulin clearances were used to measure glomerular filtration rate (GFR) and PAH clearances were used to measure renal plasma flow (RPF). Fasting blood glucose was measured to exclude hyperglycemia. If patients had diabetes, blood glucose was measured hourly. A light breakfast, allowed antihypertensive drugs and an oral water load of 5 ml/kg were given, once infusion of PAH and inulin were started (08:00 am). Thereafter, 150 ml of water was given hourly in order to maintain urine output and facilitate clearance studies. After 2 hours of equilibrium period and one hour to stabilise urine output, one hour resting clearance study was followed by hour of lower body negative pressure (−30 mbar; −22.5 mmHg). LBNP was applied with subjects in the supine position with a solid plexiglass box sealed tightly just below the iliac crests(9). Blood and urine samples were collected at the end of each hour during the clearance study. Patients remained supine for the study except for voiding, which followed blood sampling. Vital signs (BP and heart rate) were measured every 15 min during the clearance study (Omron, HEM-907, Omron healthcare Europe BV, Scorpius33, 2132 R Hoofddorp, the Netherlands).

Urinary electrolyte excretion rate was calculated as U_x_ · V and clearances (mL/min) using the standard formula C_x_ = U_x_ · V/P_x_, where U_x_ and P_x_ are the urine and plasma concentrations of x and V is the urine flow rate in mL/min. The fractional excretions were calculated as the clearance of x divided by the GFR. FF was calculated by dividing GFR by RPF. Renal vascular resistances were calculated as mean BP divided by the renal blood flow, this latter being calculated from renal plasma flow and hematocrit. BMI was calculated as weight (kilograms) divided by squared height (meters squared). Estimated GFR was calculated using the CKD-EPI equation (13).

Plasma and urinary inulin and PAH were determined by photometry (Autoanalyzer II-Technicon; Bran and Luebbe, Norderstedt, Germany). Endogenous trace lithium, used a marker of renal proximal sodium reabsorption, was measured by atomic absorption spectrophotometry (14). Plasma renin activity was measured by radioimmunoassay of generated concentration of angiotensin I using a commercial kit (GammaCoat [125I] Plasma Renin Activity Radioimmunoassay Kit, DiaSorin Inc., Stillwater, MN, USA), while measurement of aldosterone in the blood was performed with a commercial RIA kit (Aldo-Riact; CIS Bio International, Yvette. Cedex, France). Plasma catecholamines (norepinephrine and epinephrine) were measured using ultra high performance liquid chromatography-tandem mass spectrometry(15).

To compare the baseline hormonal profile and sodium excretion of patients with resistant hypertension, a group of healthy volunteers and a group of obese hypertensive patients were used. Both groups had been taking candesartan 16 mg po per day for one week and were studied in the same baseline study setting.

### Statistical Analysis

Data are expressed as mean ± standard deviation (SD) for normally distributed data and as median and interquartile range (IQR) or geometric mean with 95% CI for non- normally distributed data. Differences between variables before and after LBNP were tested using a paired t-test for normally distributed data and using a Wilcoxon matched-pairs signed-rank test for non-normally distrusted variable. Resting state PRA between resistant hypertensive participants and hypertensive obese or healthy participants was compared using a Wilcoxon rank-sum test . Resting state variables over the three phases (M0, M1, M12) were zero-skewness log or Box-Cox transformed when necessary to ensure the variable normality. Changes induced by LBNP over phases (M0, M1, M12) were examined using random intercept mixed-effect linear regression model with only the phase as explanatory variable.

## Results

Twenty-five out of ninety-six patients referred for RND were eligible and underwent the RDN procedure. Two patients included in the final analysis reported hypotensive symptoms in the week following the procedure and had their antihypertensive treatment reduced. One patient developed a pseudo-aneurysm of the right femoral artery treated by surgical repair. One patient had a microdissection of the right renal artery that was treated conservatively. Echodoppler of the right renal artery was normal after 24 h. No renal artery stenosis was detected among the patients by a systematic screening with a CT angiography after one year. Fourteen patients of the twenty-five patients eligible for RDN accepted the current study and signed the informed consent. The LBNP technique was applied to all but one participant who felt uncomfortable in the plexiglas box on the first study day, leaving 13 patients for the analysis. The Symplicity Flex catheter was used in 11 patients and the EnligHTN IV catheter in 2 patients. The mean number of ablation points in these patients was 5 [interquartile range (IQR): 4,5] points of the left renal artery and 5 (IQR: 4;5) points on the right renal artery. The total number of ablation points was 10 (IQR: 8; 10) points. No hypotension or pre-syncope signs or symptoms were noted during the LBNP period. Baseline characteristics of the patients are presented in Table 1. No change in the use of antihypertensive drugs was apparent between M0 and M12. When diuretics were grouped, the percentage of use was decreased at M1, but not at M12 compared to M0 [*p* = 0.023 (overall test), *p* = 0.012 (M1 vs. M0) and *p* = 0.790 (M12 vs. M0)].

**Table 1:** Characteristics ofthe patients before and one year after renal denervation.

Characteristics of the patients	M0	M1	M12	P (M0 vs M12)
Number (n)	13	13	13	
Age (y)	56.1 ± 9.9	-	-	-
Women (%)	15.4	-	-	-
Diabetes (%)	23.1	-	-	-
Systolic blood pressure day (mm Hg)	157 ± 23	153 ± 23	146 ± 20	0.23
Diastolic blood pressure day (mm Hg)	95 ± 12	89 ± 17	91±12	0.46
Systolic blood pressure night (mm Hg)	140 ± 24	144 ± 27	134 ± 15	0.496
Diastolic blood pressure night (mm Hg)	85 ± 12	81 ± 15	83 ± 11	0.731
BMI (Kg/m^2^)	30.9 ± 6.3	-		-
eGFR (ml/min/1.73m^2^)	69.5 ± 21.6	74.3 ± 23	65.2 ± 25.3	0.641
Number of antihypertensive drugs*	4.8 ± 1.8	3.1 ± 1.6	3.7 ± 1.7	0.103
Angiotensin II receptor blocker (%)	61.5	61.5	69.2	0.68
Angiotensin converting enzyme inhibitor (%)	23.1	7.7	7.6	0.277
Thiazide diuretic (%)	61.5	30.7	53.8	0.691
Loop diuretic (%)	30.8	30.8	38.4	0.68
Calcium channel blocker (%)	92.3	84.6	84.6	0.539
Beta-blocker (%)	69.2	53.9	46.2	0.234
Other antihypertensive (%)	76.9	38.5	61.5	0.395
Sodium excretion (mmol/24 h)	154 ± 95	190 ± 117	144 ± 21	0.839

Values are presented as mean (SD) or percentages. –, Not applicable; M0, before renal denervation; M12, 12 months after renal denervation; P, p value; BMI, body mass index; eGFR, estimated glomerular filtration rate.

* The daily number of antihypertensive drugs outside the study day at screening and after one year (M12).

### Effects of RDN in Standard Non-Stimulated Conditions

**Figure 2:**
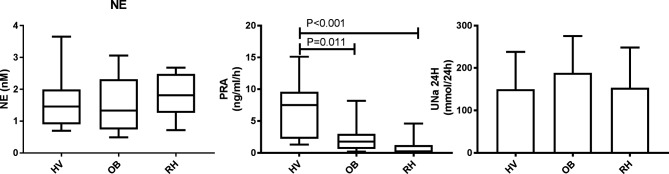
Norepinephrine (NE), Plasma renin activity (PRA) and twenty-four hour sodium excretion (UNa 24H) in healthy normotensive volunteers (HV, *N* = 20), obese normotensive and hypertensive patients (OB, *N* = 20), and in hypertensive resistant patients (RH, *N* = 13). NE, PRA and UNa 24H were measured in the same experimental setting. Data are expressed as median and interquartile range, minimum and maximum **(A)** and mean ± SD **(B)**.

Plasma norepinephrine and epinephrine levels were not different before and after renal denervation (Table 2). Both resting state PRA and aldosterone were low in these patients. PRA was much lower in patients with resistant hypertension than in healthy volunteers or in obese patients in the same experimental setting (Figure 2). In standard non-stimulated conditions, baseline PRA was also similar before and after RDN. Plasma aldosterone levels were lower one month after RDN compared to pre-RDN values but no difference was seen at 12 months.

**Table 2:** Hormones before andafter renal denervation at rest and during LBNP.

Variable	Period	Before LBNP	LBNP	*P*
Norepinephrine (nM)	M0	1.68 (1.32; 2.13)	1.91 (1.53; 2.40)	*0.039*
	M1	1.63 (1.30; 2.05)	1.98 (1.66; 2.38)	*0.055*
	M12	1.81 (1.42; 2.32)	2.02 (1.67; 2.44)	*0.279*
	*mixed model*	*P = 0.666*		
	*M1 vs. M0*	*P = 0.823*		
	*M12 vs. M0*	*P = 0.519*		
Epinephrine (nM)	M0	0.099 (0.057; 0.171)	0.167 (0.104; 0.266)	*0.015*
	M1	0.063 (0.033; 0.120)	0.110 (0.061; 0.197)	*0.208*
	M12	0.095 (0.053; 0.172)	0.110 (0.055; 0.224)	*0.624*
	*mixed model*	*P = 0.342*		
	*M1 vs. M0*	*P = 0.192*		
	*M12 vs. M0*	*P = 0.939*		
PRA (ng/ml/h)	M0	0.234 (0.095; 0.575)	0.207 (0.102; 0.421)	*0.84*
	M1	0.279 (0.117; 0.664)	0.291 (0.124; 0.685)	*0.885*
	M12	0.525 (0.213; 1.30)	0.609 (0.246; 1.51)	*0.181*
	*Mixed model*	*P = 0.236*		
	*M1 vs. M0*	*P = 0.780*		
	*M12 vs. M0*	*P = 0.111*		
Aldosterone (pg/ml)	M0	83.3 (53.9; 112.7)	72.3 (44.0; 118.8)	*0.753*
	M1	43.7 (30.6; 62.5)	42.2 (34.0; 53.0)	*0.65*
	M12	66.5 (37.8; 116.8)	60.6 (35.1; 104.3)	*0.162*
	*Mixed model*	*P = 0.038*		
	*Baseline vs. M1*	*P = 0.021*		
	*Baseline vs. M12*	*P = 0.833*		

Results are geometric means with 95% confidence interval. PRA, plasma renin activity; M0, before renal denervation; M1, 1 month after renal denervation, M12: 12 months after renal denervation

**Figure 3:**
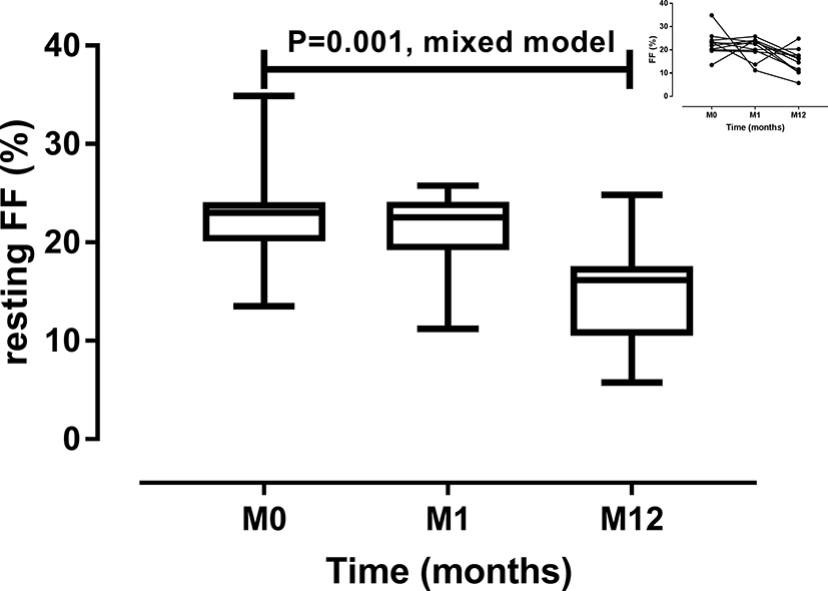
Resting filtration fraction (FF) before renal denervation (M0), 1 month (M0) and 12 months (M12) after renal denervation. Data are median, interquartile range, minimum and maximum. The small header shows individual changes.

RDN had no effect on resting systolic or diastolic BP as shown in Table 3. Resting heart rate increased from 58 ± 11 bpm (beats per minute) to 63 ± 11 bpm after one month and 64 ± 12 bpm 12 months after RDN. Baseline measured GFR but not estimated GFR (Table 1) decreased 12 months after RDN. RPF tended to increase after RDN. These changes resulted in a significant decrease in filtration fraction (FF), especially 12 months after RDN (Figure 3). Renal vascular resistances also tended to decrease after RDN.

**Table 3:** Hemodynamics beforeand after renal denervation at rest and during LBNP.

Variable	Period	Before LBNP	LBNP	*P *
SBP (mmHg)	M0	154 ± 23	161 ± 20	*0.036*
	M1	158 ± 23	163 ± 23	*0.008*
	M12	149 ± 18	151 ± 19	*0.320*
	*Mixed model*	*P* = 0.116		
	*M1 vs. M0*	*P *= 0.348		
	*M12 vs. M0*	*P *= 0.249		
DBP (mmHg)	M0	85 ± 11	92 ± 11	*0.002*
	M1	88 ± 13	91 ± 13	*0.024*
	M12	84 ± 11	86 ± 10	*0.069*
	*Mixed model*	*P *= 0.114		
	*M1 vs. M0*	*P *= 0.157		
	*M12 vs. M0*	*P *= 0.535		
MBP (mmHg)	M0	108 ± 13	115±11	*0.003*
	M1	111 ± 14	115±13	*0.002*
	M12	106 ± 10	108±10	*0.097*
	*mixed model*	*P* = 0.136		
	*M1 vs. M0*	*P *= 0.245		
	*M12 vs. M0*	*P *= 0.410		
HR (bpm)	M0	58 ± 11	60 ± 9	*0.134*
	M1	63 ± 11	63 ± 11	*0.706*
	M12	64 ± 12	63 ± 11	*0.453*
	*mixed model*	*P* = 0.030		
	*M1 vs. M0*	*P *= 0.054		
	*M12 vs. M0*	*P* = 0.011		
GFR (mL/min)	M0	78 ± 32	67 ± 31	*0.011*
	M1	79 ± 34	72 ± 31	*0.171*
	M12	66 ± 26	63 ± 40	*0.643*
	*Mixed model*	*P* = 0.012		
	*M1 vs. M0*	*P* = 0.903		
	*M12 vs. M0*	*P* = 0.012		
RPF (mL/min)	M0	353 ± 132	297 ± 94	*0.039*
	M1	380 ± 138	360 ± 149	*0.339*
	M12	429 ± 76	378 ± 148	*0.225*
	*Mixed model*	*P *= 0.127		
	*M1 vs. M0*	*P* = 0.459		
	*M12 vs. M0*	*P* = 0.044		
FF (%)	M0	22.6 ± 0.054	22.3 ± 0.058	*0.851*
	M1	20.9 ± 0.052	21.0 ± 0.056	*0.903*
	M12	15.1 ± 0.053	16.1 ± 0.062	*0.388*
	*Mixed model*	*P *= 0.001		
	*M1 vs. M0*	*P *= 0.378		
	*M12 vs. M0*	*P *< 0.001		
RVR (mm Hg/mL/min)	M0	0.222 ± 0.148	0.247 ± 0.086	*0.028*
	M1	0.195 ± 0.087	0.233 ± 0.144	*0.152*
	M12	0.145 ± 0.040	0.204 ± 0.14	*0.075*
	*Mixed model*	*P* = 0.113		
	*M1 vs. M0*	*P* = 0.659		
	*M12 vs. M0*	*P* = 0.045		

Results are mean ± SD or median with interquartiles, SBP, systolic blood pressure; DBP, diastolic blood pressure; MBP, mean blood pressure; HR, heart rate; RPF, renal plasma flow; GFR, glomerular filtration rate; FF, filtration fraction; RVR, renal vascular resistance; M0, before renal denervation; M1, 1 month after renal denervation; M12, 12 months after renal denervation.

The changes in sodium and lithium renal handling are shown in Table 4. Compared to pre-denervation conditions, urine output and urinary sodium excretion did not change after one and twelve months. In contrast, the clearance of endogenous lithium decreased twelve months after RDN.

**Table 4:** Urinary parametersbefore and after renal denervation at rest and during LBNP.

Variable	Period	Before LBNP	LBNP	*P*
UV (mL/min)	M0	3.99 ± 1.48	2.81 ± 1.22	*0.015*
	M1	4.39 ± 1.81	3.04 ± 1.32	*0.014*
	M12	4.11 ± 1.65	2.62 ± 1.41	*0.022*
	*Mixed model*	*P* = 0.742		
	*M1 vs. M0*	*P* = 0.451		
	*M12 vs. M0*	*P* = 0.823		
UNaV (µmol/min)	M0	326 ± 138	213 ± 95	*0.008*
	M1	403 ± 197	291 ± 157	*0.046*
	M12	285 ± 139	214 ± 127	*0.177*
	*Mixed model*	*P* = 0.056		
	*M1 vs. M0*	*P *= 0.184		
	*M12 vs. M0*	*P *= 0.285		
Clear Li (ml/min)	M0	22.6 ± 15.8	23.1 ± 15.0	*0.902*
	M1	19.8 ± 16.3	24.0 ± 15.9	*0.127*
	M12	11.4 ± 8.7	16.2 ± 15.9	*0.122*
	*Mixed model*	*P* = 0.002		
	*M1 vs. M0*	*P *= 0.284		
	*M12 vs. M0*	*P* = 0.002		

Results are mean ± SD or median with interquartiles, UV, urine output; UNaV, sodium excretion; Clear Li, endogenous lithium clearance; M0, before renal denervation; M1, 1 month after renal denervation; M12, 12 months after renal denervation.

### Effects of RDN in Stimulated Conditions

During LBNP, norepinephrine increased before RDN and one month after the procedure, but this increase was no longer significant 12 months after RDN (Table 2). Epinephrine levels also increased during the LBNP period but only before RDN. The magnitude of the changes induced by LBNP was, however, not different before or after RDN for both epinephrine (+0.08 ± 0.10 nM at M0, +0.06 ± 0.13 nM at M1 and +0.05 ± 0.12 at M12, *p* = 0.461 overall test) and norepinephrine (+0.25 ± 0.59 nM at M0, +0.33 nM at M1 and +0.14 at M12, *p* = 0.550 overall test). LBNP had no effect on PRA but the magnitude of the changes induced by LBNP tended to increase with time (−0.32 ± 1.12 ng/ml/h before RDN vs 0.01 ± 0.17 ng/ml/h after one month vs. 0.2 ± 0.49 ng/ml/h after 12 months, *p* = 0.061 (overall test), *p* = 0.386 (M1 vs. M0) and *p* = 0.019 (M12 vs. M0)). LBNP had no effect on aldosterone at M1 and M12 and no difference could be detected in LBNP-induced changes in aldosterone before and after RDN.

**Figure 4:**
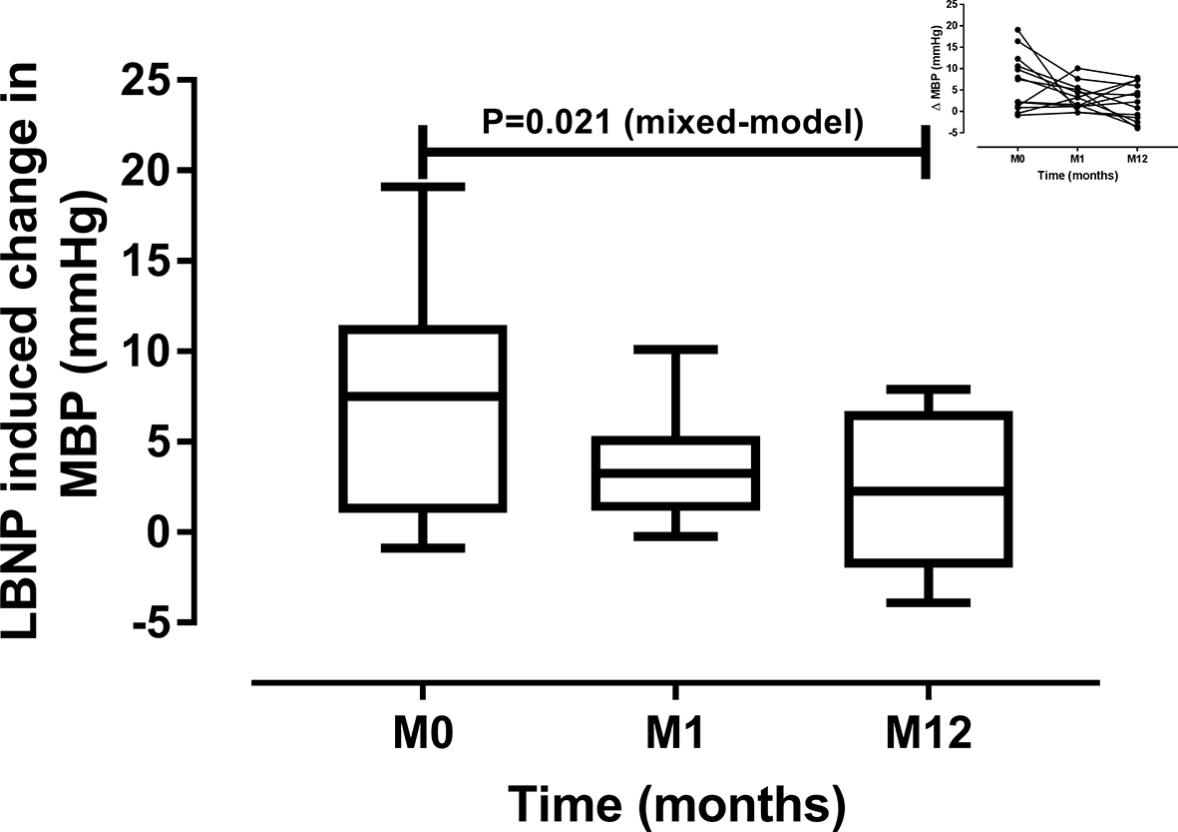
Changes in mean blood pressure (MBP) induced by lower body negative pressure before RDN (M0), 1 month (M1) and 12 months (M12) after renal denervation. Data are median and interquartile range, minimum and maximum. The small header shows individual changes.

LBNP increased SBP and DBP before RDN and one month after RDN but not at 12 months (Table 3). The magnitude of the changes in mean BP induced by LBNP diminished significantly with time as shown in Figure 4. No LBNP-induced heart rate changes were detected and there were no difference in the magnitude of the changes between baseline and following visits. During LBNP, GFR and RPF decreased significantly only before RDN. LBNP did not affect filtration fraction at any study visit.

Urine output decreased significantly during the LBNP period, before as well as 1 and 12 months after RDN (Table 3). LBNP induced a reduction of urinary sodium excretion before RDN and one month after the procedure but this effect was not seen at 12 months. The changes in lithium clearance induced by LBNP stimulation were similar before and after RDN.

## Discussion

This study is the first to focus on the neuro-hormonal, renal hemodynamic and sodium handling changes induced by RDN, not only in the resting state but also in response to an orthostatic stress induced by LBNP. Twelve months after RDN, no changes in office and 24 h BP were observed but measured GFR, using inulin clearance, was decreased and renal plasma flow increased leading to a significant reduction of filtration fraction at one year. The increase in norepinephrine induced by LBNP was blunted after RDN. This might explain why BP, GFR and RPF were less or not sensitive to LBNP after RDN. Regarding renal tubular function during orthostatic stress, the sodium reabsorption induced by LBNP was also blunted at 12 months. The results from our study also confirms that in non-stimulated conditions, patients with resistant hypertension have a down-regulated RAAS with a low renin activity and a low aldosterone, which is not stimulated by LBNP.

BP has been the primary outcome of most of the trials in RDN. The absence of effect on baseline BP, whether office or ambulatory BP (Tables 1,2), is in line with the SIMPLICITY-3 trial (2). The small sample size, not powered for ambulatory BP in our study, may also explain the heterogeneous BP response, a common finding in RDN studies (3). One original observation of the present study is that the BP response to orthostatic stress using LBNP is significantly blunted after RDN suggesting an impaired hemodynamic reactivity to stress. To our knowledge, this effect of renal denervation has never been reported. Using a tilt test, Lenski et al. found that in RDN responders, the SBP response was decreased in the initial phases of the test (16). This observation is in line with ours but one has to mention that in our study setting, the orthostatic stress was applied for one hour, i.e., much longer than a tilt test. Similarly, the reactivity of BP to exercise seems to be affected by RDN, as shown by Ewen et al (17). This group demonstrated that at mild to moderate exercise intensity, BP response was reduced 6 and 12 month after RDN. This blunted BP response to orthostatic stress using the tilt test or LBNP and to an exercise stress test suggest that even in the absence of marked decrease in BP, RDN has an impact on BP regulation and this may explain the decrease in variability of 24 h BP observed in some studies (18–20).

The use of inulin and PAH clearances allowed us to have a precise and absolute evaluation of the impact of RDN on renal hemodynamic not only in the resting state but also during orthostatic stress. The study is the first to report a decrease in measured GFR, not apparent in estimated GFR. So far, GFR estimated with the CKD-EPI creatinine based equation or with cystatin was not found to decrease after RDN (1, 2, 4, 21). Increases in estimated GFR has even been reported in patients with chronic kidney disease, in particular if BP was well controlled after RDN (22, 23). Interestingly, a post-hoc analysis of the DENERHTN trial recently reported that the degree of abdominal calcifications was associated with the BP and the estimated GFR response to RDN (24). The decrease in measured GFR using the gold standard method is important and may raise concerns about a possible unfavorable effect on renal function. It should be interpreted in the light of the increase in renal plasma flow, resulting in a significant decrease in filtration fraction 12 months after RDN and the decrease in calculated renal vascular resistance. The decrease in renal vascular resistance is consistent with the results of Ott et al., who also found a renal vasodilation after RDN using MRI with arterial spin labeling to measure renal perfusion.(21) Using Doppler ultrasound, Mahfoud et al. showed that the renal resistive index decreased three and six months after RDN in 88 patients. Patients with resistant hypertension from our study appear to have an increased renal sensitivity to orthostatic stress before renal denervation as significant decreases in GFR and RPF were observed during LBNP. This effect was also observed in healthy volunteers taking an angiotensin receptor blocker but not with a placebo(25). This vasoconstrictor effect of LBNP was blunted after RDN, pointing to a change in the renal autoregulation of blood flow. This change could be secondary myogenic or tubulo-glomerular feedback mechanisms that may also affect the renal sodium excretion. The absence of compensatory hemodynamic mechanisms during stress observed after RDN could raise safety issues, particularly with severe hypotension, such as following acute hemorrhage (26).

In our study, resting state sodium excretion was not affected by RDN, although clearance of endogenous lithium was decreased 12 months after RDN, which suggests an increased reabsorption of sodium in the proximal tubule. This effect may be the consequence of the decrease in filtration fraction. It is unlikely to be attributable to the use of diuretics, as they were prohibited on the study day. Yet, one cannot exclude that it represents a compensatory mechanism after withdrawal of diuretics(27). Moreover, the use of diuretics after renal denervation did not increase with time, but rather decreased shortly after RDN (M1). Interestingly, the orthostatic stress induced an anti-natriuresis before RDN but not after denervation. Indeed, the anti-natriuretic effect of LBNP found before RDN and at M1 was not found 12 months after RDN, suggesting that the ability to retain sodium, when kidneys are stressed, is also blunted after RDN. This effect parallels the blunted renal hemodynamics response to LBNP, which could be one of the explanatory mechanism. A direct inhibitory effect of RDN on the alpha-1 adrenoreceptor mediated sodium retention could be another explanation (7). However, the decreased lithium clearance seen 12 months after RDN, suggesting a proximal sodium retention, goes against this mechanism. Finally, other vasoactive peptides interacting with the SNS such as endothelin could have influenced the renal hemodynamic or natriuretic response (28). The clinical impact of this observation, suggesting that the pressure natriuresis curve is shifted, is not known but this phenomenon could contribute to a deterioration of renal function in acute hemodynamic stresses when salt preservation is necessary.

We did not find any change in the resting state norepinephrine levels in our patients before or after RDN. Resting state norepinephrine levels were not different from healthy volunteers or obese hypertensive patients (Figure 2). In contrast, renal norepinephrine spillover was reduced in ten patients studied in the SIMPLICITY-1 trial (1, 29). This difference could be explained by the limits of measuring peripheral norepinephrine, which depend on total regional NE release, metabolism, reuptake and clearance. Another possible explanation is that the magnitude of RDN was not sufficient to induce an observable decrease in peripheral norepinephrine. In this respect, the assessment of the procedural success has been at the heart of the debate and criticisms that followed the publication of the SYMPLICITY-3 trial (30). Interestingly, we found that one year after RDN, the increase in norepinephrine induced by LBNP was no longer significant compared to resting state values. This finding suggests that the reactivity of the SNS is blunted after RDN. It could also explain why BP, especially diastolic, measured GFR and RPF were less sensitive to LBNP one year after RDN.

The down-regulated RAAS found in our study is in line with previous observations, which have suggested that intravascular volume is increased in patients with resistant hypertension (31–33). This finding is striking since patients were given an angiotensin receptor blocker, which could explain a low plasma aldosterone but not a low PRA, which should be increased secondary to the interruption of the angiotensin II feedback loop. Indeed, PRA was much lower in patients with resistant hypertension than in healthy volunteers or in obese patients in the same experimental setting (Figure 2). Moreover, PRA was not stimulated by LBNP even if a decrease in RBF was observed before RDN, which should have increased PRA (34). A low PRA was also found in the PATHWAY-2 study despite the use of blockers of the renin angiotensin system and diuretics (33). Possible mechanisms explaining the suppressed RAS in these patients could be secondary to a high sodium intake (154 ± 96 mmol/24 h) or the decreased GFR of some of the participants. The down-regulated RAAS might explain why strategies aiming at increasing renal sodium excretion such as the use of aldosterone antagonists are effective in patients with resistant hypertension (33, 35).

The sample size of our study and the absence of a sham control group are limitations in our study. The sample size corresponds, however, to the sample size used in our previous studies of renal hemodynamics using LBNP (9, 25). The repeated measurements one and twelve month after RDN using a mixed model analysis allowed us to analyze tendencies. The constraints of the study, which include the clearances studies, the neuro-hormonal profile and the use of LBNP, also limited the number of participants included in this mechanistic exploratory study. In the absence of a control group, we cannot exclude a regression to mean effect or a spontaneous evolution of our patients. We did not however observe the development of tolerance to LBNP affecting renal or neuro-hormonal variables in our previous studies (9, 25). The mean number of ablation points may seem low nowadays compared to the more recent RDN studies (5). However, clinical evidence that increasing the number of site will improve the efficacy of renal denervation in terms of blood pressure reduction is coming for a post-hoc analysis (30). Indeed, the magnitude of 24 h BP reduction in the latest sham controlled study (mean ablation points: 43.8 ± 13.1) was not strikingly different from earlier studies with less ablation points (2, 4). Considering this issue, our data suggest an impaired renal response to stress, even with a lower number of ablation points. A more complete sympathetic nerve ablation might potentially cause an even greater impairment of the renal response to stress. Finally, the inclusion of a patient treated with insulin for his diabetes may have introduced a confounding effect on sodium handling (36, 37). The exclusion of this patient in the statistical analysis did not change the results.

In conclusion, this study shows that even in the absence of major changes in systemic BP, RDN induces a decrease in measured GFR and filtration fraction. Moreover, RDN appears to have a significant impact on the renal response to stress blunting not only the norepinephrine response but also the renal hemodynamic and the antinatriuretic responses. Whether these renal consequences of RDN will affect long-term BP and renal function remains to be evaluated in larger groups of patients.

## Ethics Statement

This study was carried out in accordance with the recommendations of the Commission cantonale d'éthique de la recherche sur l'être humain (http://www.cer-vd.ch). The protocol was approved by the Commission cantonale d'éthique de la recherche sur l'être humain. All subjects gave written informed consent in accordance with the Declaration of Helsinki.

## Author Contributions

YV: collected data, wrote manuscript. EG: catecholamines analysis, revised manuscript. OM: performed RDN, revised manuscript. NV: collected data. MF: statistical analysis, revised manuscript. SQ: performed RDN, revised manuscript. MM: inulin, PAH, endogenous lithium analysis. MB: revised manuscript. GW: designed study, collected data, integrity of data, analysis of data, wrote the manuscript.

## Conflict of Interest Statement

The authors declare that the research was conducted in the absence of any commercial or financial relationships that could be construed as a potential conflict of interest.
